# Using a Miniature Stapler to Divide a Mucosal Bridge at the Anastomosis after Gastric Pull-up for Iatrogenic Tracheoesophageal Fistula

**DOI:** 10.1055/s-0039-1678755

**Published:** 2019-03-09

**Authors:** Takafumi Kawano, Oliver J. Muensterer

**Affiliations:** 1 Department of Pediatric Surgery, University Medicine Mainz, Mainz, Germany

**Keywords:** esophageal mucosal bridge, miniature stapler, iatrogenic tracheoesophageal fistula

## Abstract

We report the first use of a miniature stapler to divide a mucosal bridge at the gastroesophageal junction after complex esophageal atresia (EA) repair. A 4-year-old girl was referred to our center after treatment of EA elsewhere. On our initial enodoscopy, a large iatrogenic tracheoesophageal fistula had formed, prompting us to perform a tracheoplasty and gastric interposition. One year after recovery, she had dysphagia with solid food. Upon endoscopy, a mucosal bridge was noted at the gastroesophageal anastomosis. This bridge was divided under endoscopy using a 5 mm miniature stapler. No complications were noted. Upon follow-up, she had no more complaints with solid food. Our report shows that the mucosal bridges may cause dysphagia after EA repair and can be safely divided using a miniature stapler.

## Introduction

Esophageal atresia (EA) is a congenital disorder that affects approximately 1 in 2,500 neonates. In the follow-up of these patients even after successful reconstruction, various complications sometimes occur long-term, such as dysphagia. Mucosal bridges after EA repair have been described in the literature before, and are one of the rare complications associated with dysphagia. 1 They should be corrected by division or resection when symptomatic. Since only a very few reports have been published about this rare condition, there are no published standards of treatment so far. We herein report the first use of a miniature stapler device to divide a mucosal bridge at the cervical gastroesophageal anastomosis after gastric interposition in the setting of a case of complex esophageal atresia.

## Case Report

**Fig. 1:**
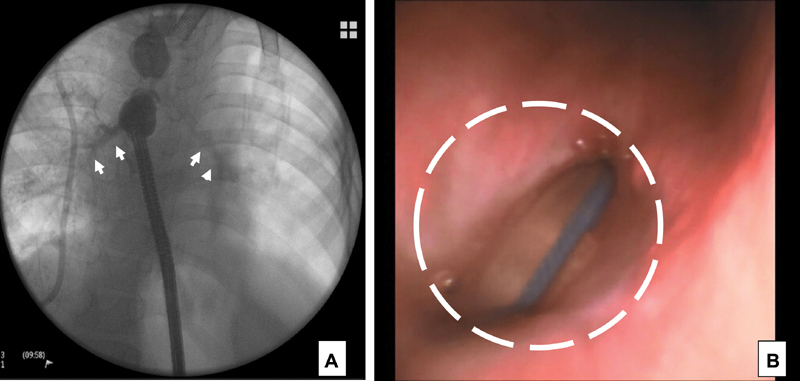
( **A** ) Upper and Lower esophagus was occuluded under endoscopy inserted through the gastrostomy, and esophagography was performed. This contrast esophagography showed tracheobronchial contrastation (arrows), raising the suspicion of an acquired tracheoesophageal fistula. ( **B** ) The endotracheal tube was identified from esophagus (circle). Tracheoscopy and endoscopy showed the large tracheoesophageal fistula, most likely due to compressive erosion by pressure from the balloon of the cuffed tracheostomy tube and the esophageal stent (circle).

**Fig. 2:**
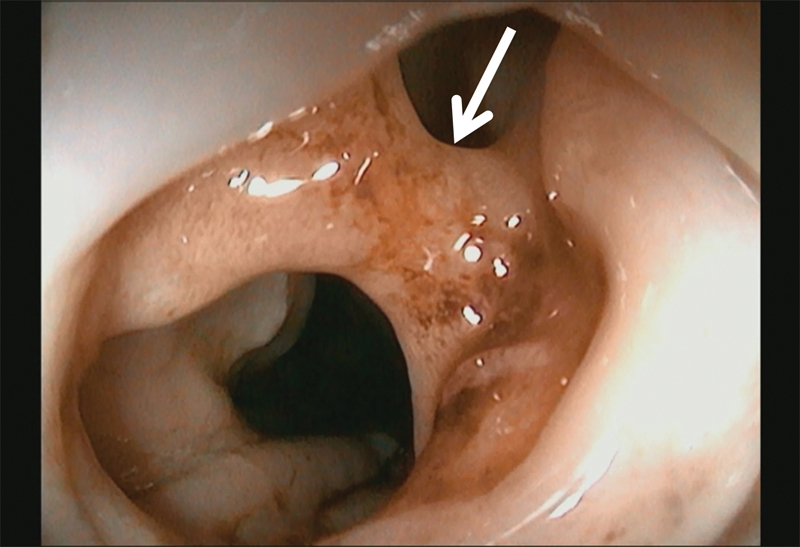
Endoscopic view of the mucosal bridge (white arrow) at the level of the gastroesophageal anastomosis.

The patient was a 4-year-old girl born at 33 weeks of gestation with EA and a distal tracheoesophageal fistula (TEF). She underwent surgical correction of EA on the early day of life. After her primary repair, she developed a multitude of complications, including anastomotic stricture and recurrent fistula. She underwent over 20 esophageal dilatations and five esophageal stent placements, as well as a tracheostomy in an outside hospital, before being referred to our center. We initially performed a bronchoscopic and esophagoscopic examination under general anesthesia to investigate the condition of esophagus. An on-table contrast esophagography showed a tracheoesophageal fistula at the level of the upper esophagus ( Fig. 1A ). Endoscopy revealed a large tracheoesophageal fistula which was most likely iatrogenic after stent placement with a cuffed tracheostomy tube in place, causing erosion of the adjacent tracheoesophageal walls ( Fig. 1B ). This finding prompted us to perform a tracheoplasty and gastric interposition using gastric pull-up procedure. After the operation, she had no symptoms and was not taking any medications, including antacid drugs. One year after recovery, the patient had dysphagia with solid food. Upon endoscopy, a mucosal bridge was noted at the level of the gastroesophageal anastomosis ( Fig. 2 ). This bridge was divided under endoscopic vision using a 6 mm flexible endoscope and a 5 mm miniature stapler (JR-ST25.2.0, JustRight Surgical) at the same time. The procedure took 25 minutes ( Video 1 ). No intra- or postoperative complications were noted. Upon follow-up, she had no more complaints with solid food.

**Video 1**

Division of a mucosal bridge using miniature stapler at the anastomosis after gastric pull-up.

## Discussion

Acquired TEF is an uncommon clinical condition, most frequently arising as sequelae to esophageal malignancy in adult patients. In addition, iatrogenic injury to the membraneous trachea secondary to cuffed endotracheal or tracheostomy tubes in the presence of an in-dwelling nasogastric tube and corrosive burns have been reported in the literature. 2 Our hypothesis for the TEF in this patient is that the combination of esophageal stent placement and cuffed tracheostomy tube caused pressure necrosis, inflammation, and erosion between these structures, forming a secondary large iatrogenic fistula. Therefore, the combination of using cuffed tracheostomy tubes and stents in the upper esophagus should be strictly avoided.

Mucosal bridge is a very rare condition and the etiology is still unclear. I can form after an anastomosis, perhaps due to injury from indwelling catheters, contained leaks, gastroesophageal reflux disease, inflammation, esophageal dysmotility, or a combination of the above. 1 The mucosal bridge of this patient may have been the result of gastroesophageal reflux from the interposed stomach into the upper pouch. Endoscopy is the method of choice to diagnose these mucosal bridges and should be performed for patients that exhibit continuous or progressive symptoms of dysphagia.

Some asymptomatic esophageal mucosal bridges were not treated. 3 However, in those showing symptoms, they should be divided or resected. Up to now, various techniques and cutting devices have been employed to dissect the bridge; including argon plasma coagulation, endoscopic knives, or endoscopic scissors. 4 Recently, treatment of an esophageal diverticulum with a 5 mm endoscopic stapler has been reported. 5 This prompted us to use the endoscopic miniature stapler on the mucosal bridge of this patient which was simple, quick (25 minutes operative time), and without complications.

However, the limitation of this technique is the size of the patient, because it is necessary to insert the 5 mm stapler and the endoscope into esophagus at the same time. In addition, an intraoperative challenge we encountered during the procedure was to obtain a good seal for gas insufflation during flexible endoscopy with both the endoscope and the stapler at the cervicothoracic junction. Therefore, rigid esophagoscopy may be an alternative that does not require insufflation and therefore may be even quicker.

## Conclusion

The combination of esophageal stent placement and cuffed tracheostomy tube can cause tracheoesophageal fistula by erosion and therefore should be avoided. Mucosal bridges are rare but may cause dysphagia after esophageal atresia repair. They can be safely divided endoscopically using a 5 mm miniature stapler.

## Notes

**New Insights and the Importance for the Pediatric Surgeon**
The combination of esophageal stent placement and cuffed tracheostomy tube can cause tracheoesophageal fistula by erosion and therefore should be avoided. Mucosal bridges are rare but may cause dysphagia after esophageal atresia repair. They can be safely divided using a 5 mm miniature stapler.
